# The moral foundations of cryptocurrency: evidence from Twitter and survey research

**DOI:** 10.3389/fpsyg.2023.1128575

**Published:** 2023-08-09

**Authors:** Sachin Banker, Joowon Park, Eugene Y. Chan

**Affiliations:** ^1^The University of Utah, Salt Lake City, UT, United States; ^2^Toronto Metropolitan University, Toronto, ON, Canada

**Keywords:** cryptocurrency, moral foundations, political ideology, conservatism, liberalism

## Abstract

Despite its relatively brief history, cryptocurrency has already had a profound impact on the economy, with some predicting that it will eventually replace traditional fiat currencies. Historically, it had dark associations with illegal activities in the early days, although perceptions and associations likely have, in recent years, changed for the better. Thus, understanding how people perceive the morality of cryptocurrency currently forms the motivation of the current research. We, in particular, examine associations dependent on political ideology. Across both a large-scale analysis of Twitter posts (*N* = 959,393) and controlled survey research (*N* = 487), we find that cryptocurrency is currently best understood as being more strongly linked to conservative vs. liberal moral foundations. Cryptocurrency-related posts were more likely to express conservative moral foundations (Authority, Purity, and Loyalty) rather than liberal moral foundations (Fairness and Care), and individual endorsement of these conservative moral foundations was associated with increased interest in crypto investment.

## Introduction

Cryptocurrency is growing around the world. Investors are attracted to potential financial gains and cryptocurrency’s technology while regulators are investigating crypto’s risks. The novel asset class also invites several questions from behavioral researchers. While there has been relatively little discussion of cryptocurrency in the consumer psychology literature, one important question that has important implications for both developers of crypto-based projects and regulators is the characteristics of investors who are attracted to cryptocurrency. In previous research by Martin et al. (2022), empirical findings have shown that personality traits of Machiavellianism, narcissism, psychopathy, and sadism (known as the Dark Tetrad) are associated with interest in cryptocurrency. The current research builds on this approach to investigate crypto advocates from a related perspective that has been closely associated with crypto space since the dawn of the space: morality.

The morality of the crypto community has been questioned since its beginnings in 2009 due to several events that made the headlines. Historically, cryptocurrency has been associated with illegality and thus “immoral” actions. A quick Internet search would give readers plenty to explore about some morally questionable players and events associated with the crypto space. Yet, more recently, the morality of cryptocurrency has likely been normalized with wider adoption among people across the world. Consequently, it is not clear what moral principles are associated with the cryptocurrency space at present. What are the moral underpinnings embodied by the crypto community today? What aspects of morality predict interest in investing in cryptocurrency? Recent discussions have noted that both conservatives and liberals, groups shown to have differing moral foundations, can adopt crypto technology to advance their ideals (The Economist, 2022), but currently little is known about the actual moral values held by the crypto advocates. We address these questions in this research through the combined analysis of a large text corpus of Twitter posts and a controlled survey study. Our goal is to provide a snapshot of the moral characteristics of crypto advocates at the current stage of adoption with the hope that our findings lay the groundwork for future research in further investigating underlying mechanisms and the applications of the current findings.

One framework to understand people’s perceptions of actions and behaviors involves examining one’s moral intuitions. Namely, Moral Foundations Theory (MFT) proposes that individuals make judgments about proper behavior, “approval versus disapproval,” and “right versus wrong,” based on six moral intuitions (recently expanded from the five foundations originally proposed) (Graham et al., 2009; Atari et al., in press). MFT proposes six moral foundations along which proper behavior is intuitively evaluated against: Care (cherishing and protecting others), Equality (equal treatment and equal outcome), Proportionality (reward proportional to one’s contribution), Loyalty (standing with one’s group), Authority (following established rules and promoting stability), and Purity (abhorrence for what is unnatural). Our research thus seeks to examine which set of moral foundations predict interest in cryptocurrency. In the following section, we describe why Moral Foundations Theory is particularly relevant in understanding crypto investor characteristics and discuss implications for crypto projects and politicians.

## Theoretical framework

### Morality of cryptocurrency

Historically, public perceptions of cryptocurrency were influenced by a series of scandals that likely contributed to negative moral evaluations of the technology and its users. For example, the first well-known platform on which Bitcoin was used as a form of payment was an online marketplace named “Silk Road” where people bought and sold illicit items such as narcotics and forged passports. Other early platforms adopting Bitcoin payments, such as SatoshiDice, were also used for unregulated gambling (Popper, 2015). In addition, a number of crypto services and projects have been revealed to be fraudulent attempts to scam users out of their financial investments. As a consequence, many public figures hailed cryptocurrencies as morally-dubious projects, such as Janet Yellen, the U.S. Secretary of the Treasury, expressing her view in January 2021 that cryptocurrencies are used “mainly for illicit financing” (De, 2021). Lay perceptions of the cryptocurrency community that were formed based on these historical events may generally associate it with negative moral values.

However, more recent developments may have changed this perception. Wider adoption as well as further efforts to educate people about the benefits of blockchain technology may have helped create more positive perceptions. For instance, Janet Yellen more recently in March 2022 expressed that the cryptocurrency space has “grown by leaps and bounds” and “there are benefits from crypto and we recognize innovation in the payment systems can be a healthy thing.” Some recent examples help to illustrate how cryptocurrencies may be used to facilitate more virtuous activities. For instance, adoption of crypto payment systems has grown among millions of workers working in foreign countries who send money home to their families in their home countries in order to avoid exorbitant fees typically demanded by traditional financial institutions. In addition, Ukrainian refugees fleeing their home country were provided with a means to transport their wealth without requiring potentially less reliable centralized financial institutions. Recent research also demonstrated that bitcoin-denominated pricing of products can actually reduce preference for vice goods (Park and Banker, 2022). As the cryptocurrency space evolves and expands its community, what moral values do crypto advocates now exhibit?

In short, while cryptocurrency was historically viewed by laypeople as a tool that facilitated immoral actions of its users, new use cases and wider adoption have potentially changed the moral values associated with the community. Such complexity leaves us with an important question: Who are retail investors interested in investing in cryptocurrency and what are their moral characteristics? The current research sets out to answer this question with an exploratory approach that provides a snapshot of the moral values of individuals who are interested in investing in cryptocurrency at the current stage of adoption.

### Moral foundations theory

One framework to understand people’s attitudes toward certain behaviors and actions is by examining the moral intuitions underlying those attitudes. Namely, Moral Foundations Theory (MFT) (Graham et al., 2009, 2011; Strimling et al., 2019; Waytz et al., 2019) examines how people make judgments about proper behavior and “right versus wrong” and has recently been applied toward understanding consumer behavior (Chan and Meng, 2020; Hang et al., 2021; Tal et al., 2022). MFT originally proposed five central moral foundations along which proper behavior is intuitively evaluated against: Care involves intuitions that prevent harm and caring for others; Fairness produces intuitions involving reciprocity and justice; Loyalty involves intuitions relating to sacrificing for one’s in-group; Authority is associated with intuitions that respect for and obedience to authority figures, social traditions, and hierarchies; and Purity emphasizes bodily and moral purity in contrast to degradation. Some classifications have grouped Care and Fairness into a single “individualizing foundation” and Loyalty, Authority, and Purity into the “binding” foundations (Graham et al., 2011; Haidt, 2012). Furthermore, in recent literature, Fairness has been subdivided into the two distinct foundations of Equality and Proportionality, resulting in updated MFT measures that include six foundations (MFQ-2; Atari et al., in press). Here, Equality refers to equal treatment and outcomes (e.g., everyone receives the same share of the pie), while Proportionality reflects a dependence on one’s contribution (e.g., contributions of 1 h receive 1 unit; contributions of 2 h receive 2 units). We apply both operationalizations, the original MFT with five foundations and the more recently expanded MFT with six foundations across our studies.

Prior research has shown that the moral foundations against which people innately evaluate “proper behaviors” predict a host of behavioral outcomes. For instance, individuals who place greater value on the Purity foundation are more hesitant to use vaccines for children (Amin et al., 2017; Hornsey et al., 2018; Rossen et al., 2019). Meanwhile, individuals who place value on the Care and Fairness foundations are more likely to donate money to charity (Winterich et al., 2012; Nilsson et al., 2016). Moreover, the Fairness foundation has been suggested to predict support for punishment in crimes involving sexual aggression (Harper and Harris, 2017). Transgression of different moral foundations has been shown to engender different emotional reactions in observers (Cannon et al., 2011). In all of these and other cases, people intuitively evaluate a behavior or judgment along the relevant moral foundations, coming to a formal assessment of what to do (or not do).

Why does the current research specifically focus on the moral foundations of crypto advocates instead of studying a myriad of other personality variables? Moral foundations theory offers an important benefit—namely, people’s underlying moral intentions can strongly predict attitude-behavior consistency. That is, when individuals rely on a moral foundation for the basis of an attitude, the attitude more strongly predicts behavior (Skitka et al., 2005; Skitka and Bauman, 2008) and is more resistant to change (Aramovich et al., 2012). This offers an important reason to explore the moral foundations underlying attitudes toward (and behaviors involving) cryptocurrency over other personality variables. Once researchers understand the moral basis for people’s attitudes toward cryptocurrency, it offers a stronger basis to predict actual behavior (e.g., cryptocurrency use or investment). To be sure, why attitudes with a moral basis better predict behavior is theoretically unclear. Some research offers the possibility that such attitudes are more stable and internal (Rozin, 1999) while others offer genetic reasons (Brandt and Wetherell, 2012). Either perspective implies that attitudes grounded on a moral basis are also more stable and resistant to change.

One important domain that has been often examined in conjunction with MFT is political orientation. It is well-established that Care and Fairness can be subsumed as “individualizing” foundations, while Loyalty, Authority, and Purity are subsumed as “binding” foundations. Related to political ideology, while both liberals and conservatives place a similar emphasis on the individualizing foundations, conservatives value binding foundations more so than liberals (Graham et al., 2009; Winterich et al., 2012; Day et al., 2014). Thus, given how political beliefs might predict reliance on one foundation or another (or one set of foundations or another), understanding the moral foundations of crypto people offers further insight in determining *who*, depending on their political beliefs and attitudes, are drawn to cryptocurrency.

### Political ideology

Political ideology represents a range of competing philosophies about life and how it should be lived (Jost et al., 2009). Political ideology is also relevant in purchasing and investment contexts, where the individual’s political ideology often plays a pivotal role in shaping their brand-related attitudes, opinions, and behaviors (Jung et al., 2017; Chan and Ilicic, 2019; Arli et al., 2022; Cui and van Esch, 2022; Harnish et al., 2022; Shewani and Chan, 2022). This comes about because contemporary consumption is a “primary arena in which political ideology is expressed and constructed” (Crockett and Wallendorf, 2004, p. 511). Part of this may stem from the fact that all people hold *some* sort of political ideology, and ideology can influence behavior outside the voting booth by offering a “lens” through which to see the world. As a result, the influence of political ideology on individual attitudes and behaviors has been observed across a wide range of contexts.

There are numerous ways to distinguish between people who hold a conservative ideology from those with a liberal one (Bafumi and Shapiro, 2009; Khan et al., 2013), two of which tend to be most primary and well-established (Thorisdottir et al., 2007; Jost et al., 2017). On social matters, conservativism is related to traditional and historically or socially accepted values and customs. On fiscal matters, conservatism is linked to hierarchy, even if it means economic disparity among individuals within a society. As examples, conservatives are more opposed to homosexuality and abortion as they go against historically or socially accepted practices (social dimension), and they are more opposed to public health care and social welfare as such policy objectives aim to reduce inequality (fiscal dimension). The different lens through which to view the world can explain ideological differences across many domains such as self-control, happiness, and health (Napier and Jost, 2008; Clarkson et al., 2015; Chan, 2019).

As mentioned above, MFT has often been applied to understand why moral judgments vary across the political spectrum, such as in understanding the “culture wars” between political liberals and conservatives in the U.S. (Haidt and Graham, 2007). The prior research has shown that political liberals tend to score higher on Care and Fairness foundations (i.e., individualizing foundations), while political conservatives instead tend to score higher on Loyalty, Authority, and Purity foundations (i.e., binding foundations) (Graham et al., 2009; Winterich et al., 2012; Day et al., 2014; Kivikangas et al., 2021).

Given the close association of moral foundations to political orientation and the importance of one’s political orientation in determining one’s attitudes, opinions, and behaviors, another question we seek to examine in this research is whether cryptocurrency is more closely linked to the moral foundations of political liberals or conservatives. The answer to this question is not unequivocal. It is evident in the media and public discourse that there are different attitudes toward cryptocurrency among people of different political ideology—especially among participants of different ideologies within the United States. For example, Republican lawmakers tend to be supportive of cryptocurrency and especially Bitcoin because of its ability to create new jobs, while Democratic lawmakers are also on the whole appreciative of the job growth opportunities that come with crypto yet are concerned with the potential environmental effects of digital asset mining (Hamilton, 2022; Mak, 2022). Some Republican lawmakers have even gone so far to protect cryptocurrency investments in 401(k) accounts (Henney, 2022). There are also similarities in attitudes toward cryptocurrency worldwide, such as the left-leaning Labor government in Australia seeking to regulate crypto more (Markezic and Bacina, 2022), yet federal conservative party leader Polliviere promotes Bitcoin in Canada (McGregor, 2022) and right-leaning French president Macron seeking to protect the new digit asset class by introducing tax-exempt policies (Zhuang, 2022). Consequently, there does seem to be different attitudes toward cryptocurrency held by members holding diverging political ideologies and the current research seeks to provide the first behavioral scientific observation on this topic.

## The current research

In this research, we examine the moral foundations that are currently exhibited by people interested in cryptocurrency. Specifically, what are the moral foundations of individuals who are interested in purchasing and utilizing cryptocurrency? Are these individuals higher on some foundations and lower on others? Building on these findings, can we understand how the political orientation of investors is related to their interest in cryptocurrency?

We address these questions in two studies. As an initial exploration, in Study 1, we first analyze the moral foundations expressed in language on social media by scraping a large volume of posts from the crypto community made on Twitter. Next, in Study 2 we adopt a paradigm introduced in Martin et al. (2022) to conduct a survey allowing us to further understand the moral foundations and political orientation associated with crypto interest. All Twitter data are publicly available via API and survey data are available upon request.

## Study 1

### Method

#### Tweet collection and cleaning

As an initial exploration, we examined the moral language used by crypto Twitter when discussing cryptocurrency. We analyzed public tweets posted on Twitter that were related to Bitcoin (i.e., mentioning “Bitcoin,” “#btc,” or “$btc”). The tweets included only those posted by verified accounts and did not include retweets or replies. Using Twitter’s API, we scraped in total *N =* 959,393 tweets matching these criteria which spanned the time period of July 3, 2008 (the date on which first bitcoin tweet was made) to July 31, 2022 (the last day prior to our data collection). Note from the date of our data collection that we used the legacy Twitter verification criteria prior to the introduction of paid Twitter Blue service for verification. We cleaned tweets using a Python script that removed URLs and punctuation.

#### Moral-language analysis

Analyzing moral sentiment in natural language such as a large set of tweets allows researchers to gain valuable insights that complement traditional surveys (Hoover et al., 2020). We applied recent natural language processing methods to examine the extent to which crypto tweets reflect each of the five original moral foundations. This method adopts word-embedding algorithms that capture semantic similarity between words by mapping each word onto a high-dimensional space, something that cannot be done with traditional word count measures such as LIWC (Pennebaker et al., 2001). Consequently, word-embeddings allow researchers to numerically represent relationships between words and have increasingly been applied within the literature (Berger et al., 2020; DeFranza et al., 2020).

For intuition in applying this method, consider the words “pure,” “impure,” and “theft.” While it may be easy for humans to determine that “theft” is semantically closer to “impure” than it is to “pure,” how can an algorithm determine the semantic closeness and quantify it to be used for analyses? Following the Distributional Hypothesis (Firth, 1957), this method assumes words that frequently co-occur in similar contexts to have more semantic closeness than words that co-occur less frequently. This is made possible using GloVe (Pennington et al., 2014), an algorithm that maps each word onto a 200-dimensional space, where each dimension captures a distinct aspect of the word’s meaning, based on its usage in a large dataset of tweets. For each word in the pretraining dataset (i.e., 2 billion tweets), GloVe looks at the words that commonly appear around it and determines the probabilities of these co-occurrences. These probabilities are then translated into numerical values, corresponding to the 200 dimensions of the vector that place each word at a unique location in the vector space. Thus, each word’s vector is a mathematically-encoded version of its semantic context, allowing us to quantify and compare meanings across different words. The higher the dimensionality of the word embeddings, the more nuanced the semantic representation can be, with 200 being a commonly used standard in the field. We can then apply these learned representations to measure the semantic distance between focal text (i.e., tweets) and the constructs of interest (i.e., moral foundations like Purity).

In operationalizing the constructs of interest (i.e., each dimension of moral foundation), we used the distributed dictionary representation (DDR) approach. For each moral foundation, the semantic distance of focal text (i.e., tweets) is measured against a dictionary of words that represents the moral foundation (i.e., a dictionary of words similar or dissimilar to “Purity” etc.), where we applied dictionaries validated in prior research (Garten et al., 2018; Wang and Inbar, 2021). Using the words from each tweet, GloVe word-embeddings provided a 200-dimensional vector representation of the tweet, averaged across the words. Similarly, for each moral foundation dictionary, a 200-dimensional vector represented the moral foundation. To evaluate how closely aligned each tweet was to the moral foundation, we measured the cosine distance between the two vectors. The previously developed dictionaries that we applied corresponded to the original five dimensions of moral foundations (Care, Fairness, Loyalty, Authority, Purity), with further validation details reported in prior work (Garten et al., 2018; Wang and Inbar, 2021); notably, we extend this prior work by using more context-sensitive GloVe embeddings rather than word2vec. Because the Wang and Inbar (2021) dictionaries included two dictionaries for each moral foundation (“virtue” and “vice” dictionaries), after calculating cosine distance, we took the difference of these two for each moral foundation to capture the semantic similarity. For clarity, this is summarized in the equation below:


$$\begin{array}{c} \mathrm{MF}\;\mathrm{Similarity}=\mathrm{Cosine}\;\left(\mathrm{Tweet,}\;\;\mathrm{MF}\;\mathrm{Virtue}\;\mathrm{Dictionary}\right)\\ -\;\;\mathrm{Cosine}\;\left(\mathrm{Tweet,}\;\;\mathrm{MF}\;\mathrm{Vice}\;\mathrm{Dictionary}\right). \end{array}$$

where Cosine (x1, x2) represents the cosine distance function, Tweet represents the 200-dimensional average GloVe word-embedding associated with the tweet, and MFVirtueDictionary and MFViceDictionary represent 200-dimentional average GloVe word-embeddings associated with the MFT dictionaries applied in Wang and Inbar (2021).

To summarize, we employed the Distributed Dictionary Representations (DDR) procedure to ascertain the moral foundations present in tweets, in five main steps. Firstly, we pre-processed the tweets, removing URLs, special characters, and punctuation. Secondly, each word in the tweets was represented as a 200-dimensional vector using GloVe word embeddings, encapsulating the semantic meaning of the word. In the third step, we averaged these word embeddings to generate a single vector symbolizing the overall semantics of the tweet. The fourth step involved calculating the cosine similarity between this tweet vector and virtue and vice dictionary vectors for each moral foundation, thus enabling the detection of implicit word associations. Finally, we interpreted the resulting value from subtracting the cosine similarity between the tweet vector and the vice dictionary vector from the cosine similarity between the tweet vector and the virtue dictionary vector, thereby revealing the semantic alignment between the tweet and each moral foundation’s virtues and vices.

### Results and discussion

To evaluate whether Bitcoin-related tweets overall expressed moral language that was similar or dissimilar to each of the moral foundations, we tested the mean similarity against zero for each moral foundation. A positive average similarity score indicated that tweets expressed moral language that was similar in sematic meaning to the moral foundation, whereas a negative similarity score indicated that tweets expressed moral language that was dissimilar in sematic meaning to the moral foundation. A zero measure indicated no relationship.

**Figure 1:**
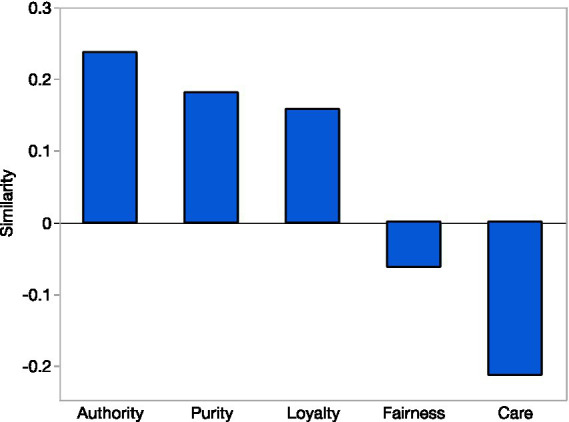
The extent to which bitcoin tweets (*N* = 959,393) represented each dimension of moral foundation measured using semantic similarity score. Y-axis displays average semantic similarity with each moral foundation.

In a sample of nearly 1 million tweets, our findings revealed that tweets expressed moral language that was positively related to the binding moral foundations of Authority (*M = 0*.236, SD *= 0*.053, *t*(959392) = 4,346, *p < 0*.001), Loyalty (*M = 0*.157, SD *= 0*.038, *t*(959392) = 4,051, *p < 0*.001), and Purity (*M = 0*.181, SD *= 0*.040, *t*(959392) = 4,432, *p < 0*.001). In addition, tweets were overall negatively related to the individualizing moral foundations of Care (*M =* −0.212, SD *= 0*.055, *t*(959392) = 3,799, *p < 0*.001) and Fairness (*M =* −0.062, SD *= 0*.030, *t*(959392) = 2002, *p < 0*.001). See Figure 1 for the graphical representation of the results.

These findings indicate that moral values expressed in language on crypto-Twitter exhibits greater semantic similarity to conservative ideals (i.e., binding foundations of Loyalty, Authority, and Purity) rather than liberal ideals (i.e., individualizing foundations of Care and Fairness).

## Study 2

Study 1 illustrated that people involved in the crypto community on Twitter tend to express language reflecting moral foundations more closely associated with conservative versus liberal ideals. In Study 2, we explored this further by conducting a survey to understand what moral foundations distinguish those who are interested vs. not interested in crypto, and the relationship to political orientation. Furthermore, to establish more generalizability, we expanded our focus from Bitcoin in Study 1 to cryptocurrency in general in Study 2.

### Methods

#### Participants

We recruited a total of 500 participants located in the United States through Prolific (preregistration link: https://aspredicted.org/PNP_KJV), of which 487 passed all attention checks and were included in the analysis (297 women, age *M* = 37.47, SD = 13.30). The sample size was set similar to that of prior closely related research examining crypto interest that we directly build on Martin et al. (2022).

#### Procedures

All participants answered a series of questions probing interest and attitudes toward cryptocurrency. Adapted from Martin et al. (2022), these questions included three items related to one’s interest in investing in cryptocurrency (e.g., “If you were looking to invest, how likely are you to buy cryptocurrency?” 1 = unlikely, 7 = likely; *α* = 0.989) and three items related to attitudes toward cryptocurrency (e.g., “How do you feel about cryptocurrency?” 1 = bad, 7 = good; *α* = 0.980). Participants also completed the MFQ-2 scale (Atari et al., in press) in order to measure moral foundations. The questions that were used to measure interest in investing in crypto and attitudes toward crypto can be found on Web Appendix. The 36-item MFQ-2 scale extends the previously developed MFQ scale (Graham et al., 2008) by separating the Fairness foundation into Proportionality and Equality foundations. The MFQ-2 instrument included six subscales corresponding to Care (Cronbach *α* = 0.913), Equality (*α* = 0.910), Proportionality (*α* = 0.835), Loyalty (*α* = 0.868), Authority (*α* = 0.903), and Purity (*α* = 0.823). Then participants shared their political affiliation by indicating which political party they support (Democratic Party, Republican Party, Libertarian Party, Other, Independent). Finally, we captured additional individual demographic differences by asking participants to share information about age, gender, education, and income.

### Results and discussion

#### Descriptive statistics

We present descriptive statistics summarizing interest in cryptocurrency, attitude toward cryptocurrency, and each of the six moral foundations in Table 1. Furthermore, we present zero-order correlations between the variables in Table 2.

**Table 1:** Descriptive statistics in study 2.

Measure	*M*	SD
Crypto interest	9.50	5.87
Crypto attitude	10.81	4.91
Care	24.32	4.77
Equality	17.02	6.70
Proportionality	22.07	4.90
Loyalty	16.03	5.94
Authority	17.50	6.35
Purity	13.86	5.84

**Table 2:** Zero-order correlations between variables in study 2.

	Crypto interest	Crypto attitude	Care	Equality	Proportionality	Loyalty	Authority	Purity
Crypto interest								
Crypto attitude	0.83**							
Care	−0.09*	−0.14**						
Equality	−0.01	−0.04	0.41**					
Proportionality	0.04	0.07	0.17**	−0.26**				
Loyalty	0.14**	0.18**	0.19**	−0.17**	0.56**			
Authority	0.15**	0.18**	0.11*	−0.24**	0.58**	0.85**		
Purity	0.16**	0.16**	0.08	−0.20**	0.46**	0.68**	0.75**	

**p < 0.01, *p < 0.05.

#### Moral foundations and crypto interest

**Figure 2:**
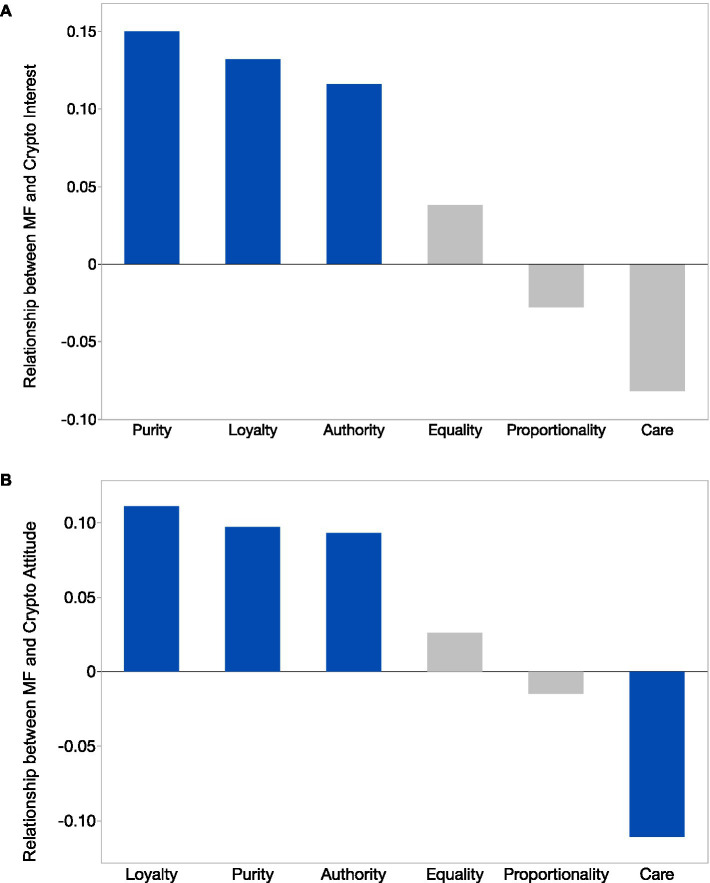
**(A)** Relationships between moral foundations and interest in cryptocurrency investment. Y-axis displays parameter estimates from regression analysis with demographic controls. Positive parameters reflect greater interest in cryptocurrency investment. Blue bars indicate significant relationship. **(B)** Relationships between moral foundations and attitudes toward cryptocurrency. Y-axis displays parameter estimates from regression analysis with demographic controls. Positive parameters reflect positive relationship. Blue bars indicate significance.

Following our preregistration plan, we conducted regression analyses to evaluate the relationship between moral foundations and interest in investing in cryptocurrency. Interest in investing in cryptocurrency was the dependent variable and regressors included demographic controls (age, gender, education, income, and political affiliation) and each moral foundation estimated separately. Our findings indicated that binding moral foundations, Loyalty (*b = 0*.132, *se = 0*.049, *t*(475) = 2.69, *p = 0*.008), Authority (*b = 0*.116, *se = 0*.046, *t*(475) = 2.51, *p = 0*.013), and Purity (*b = 0*.150, *se = 0*.047, *t*(475) = 3.17, *p = 0*.002) were positively associated with interest in investing in cryptocurrency, while Care (*b =* −0.082, *se = 0*.054, *t*(475) = 1.54, *p = 0*.125), Equality (*b = 0*.038, *se = 0*.042, *t*(475) = 0.91, *p = 0*.363), and Proportionality (*b =* −0.028, *se = 0*.055, *t*(475) = 0.51, *p = 0*.613) did not have a significant relationship. A graphical summary of these results is presented within Figures 2A,B. Consistent with our findings in Study 1, these results indicated that people interested in crypto held moral foundations that were more closely associated with conservative ideals (i.e., binding foundations of Loyalty, Authority, and Purity) than with liberal ideals (i.e., individualizing foundation of Care). It should also be highlighted that despite the difference in study methodologies, across both studies crypto enthusiasts displayed stronger association with binding moral foundations that are typically associated with political conservatives.

#### Political affiliation and crypto interest

Within our sample, 49% of participants affiliated with the Democratic Party, 18% with the Republican Party, 24% were independents, 4% Libertarian, and 4% Other. We present averages for participants who affiliated with the Democratic and Republican parties in Table 3 below. Notably, those who self-identified as Republicans scored higher on all three dimensions of binding moral foundations (i.e., Loyalty, Authority, and Purity) than did participants who self-identified as Democrats. These results are consistent with the prior findings that political conservatives value binding moral foundations more than political liberals. In addition, our results show that Republicans have more positive attitudes toward cryptocurrency and are more interested in investing in cryptocurrency than Democrats. This is important as understanding the political orientation of crypto advocates offers an actionable segmentation basis for businesses and policy makers in their targeting and message design efforts.

**Table 3:** Average measures by political affiliation, with standard deviation shown in parentheses.

Measure	Democrats	Republicans	*t*	*p*
Crypto interest	8.39 (5.55)	10.55 (5.79)	3.08	0.002
Crypto attitude	9.78 (4.61)	12.09 (4.81)	3.98	<0.001
Care	24.93 (4.63)	23.53 (4.63)	2.41	0.016
Equality	19.30 (6.05)	12.42 (5.57)	9.32	<0.001
Proportionality	20.75 (4.92)	24.53 (3.82)	6.53	<0.001
Loyalty	14.09 (5.04)	21.23 (4.88)	11.47	<0.001
Authority	14.97 (5.55)	23.11 (4.71)	12.26	<0.001
Purity	11.86 (4.81)	18.26 (6.01)	9.98	<0.001

DF = 327 in all *t*-tests.

### General discussion

Cryptocurrency is a technology that has the potential to house both conservative and liberal dreams. However, in line with viewpoints of Peter Thiel and Marc Andreessen (The Economist, 2022), our findings document convergent evidence indicating that crypto is best understood as “right-wing tech” more closely aligned with conservative moral foundations at the current stage of adoption. Our analyses of a large set of Bitcoin tweets and a controlled survey indicate that binding moral foundations (Authority, Purity, and Loyalty) that are more closely associated with political conservatives better reflect one’s interest in cryptocurrency than individualizing foundations (Fairness and Care).

While the current research focused on providing a snapshot of the moral foundations of crypto advocates at the current stage of adoption, our findings lay the groundwork for future research that digs deeper into understanding why the moral foundations of cryptocurrency are more conservative-leaning than liberal. One defining characteristic of cryptocurrency, at least on the surface, is decentralization backed by entrepreneurialism. Cryptocurrency challenges the idea that only the centralized organizations (i.e., governments and centralized banks) should have control over the regulation of money. It is possible that government interventions that led to the destabilization of economy around the globe (e.g., bailout of banks, questionable monetary policy leading to high inflation and rate hikes in response causing economic slowdown, questionable fiscal policy ballooning government debt, etc.) reduced people’s confidence in governments and led them to seek alternatives in cryptocurrency. Further research could examine whether events that reduce people’s confidence in their government are linked to increased interest in crypto Our current study focused on crypto advocates within the United States, and given the variance in cultures and governments across the world, regional differences would be valuable to explore as well. Our findings regarding the moral foundations of cryptocurrency, particularly its stronger association with conservative ideologies, have significant implications for both businesses and society. Businesses operating in or considering entering the cryptocurrency market can leverage these insights to align their strategies with the moral foundations of their target audiences, potentially leading to higher user engagement and adoption rates. Moreover, policymakers can utilize this understanding to formulate responsive and effective cryptocurrency regulations that account for the moral and political inclinations of the public, fostering a more balanced and inclusive cryptocurrency ecosystem.

This perspective unlocks several novel avenues toward understanding how individual behavior involving crypto technologies may be a function of political ideology. Literature has shown that conservatives are more prone to anthropomorphize (Chan, 2020), variety seeking (Fernandes and Mandel, 2014), avoid ambiguity (Farmer et al., 2021), among other cognitive and motivational biases (Jost, 2017). These tendencies could help to identify vulnerabilities to predatory scams and marketing activities that are more predominant within crypto communities than traditionally studied financial decision making contexts. It would also be insightful to consider the intensity of political inclination as a potential moderating factor in the relationship between moral foundations and attitudes towards cryptocurrency. Crypto projects seeking to increase adoption could take advantage of the malleability of preference for individualizing and binding foundations (Napier and Luguri, 2013). Furthermore, our findings that crypto investors hold stronger binding foundations (Loyalty, Authority, and Purity) point to messaging strategies that policymakers can leverage in the design of more effective warnings and risk communications (Kidwell et al., 2013).

One may wonder why “honesty” was not studied in research that tries to understand the moral characteristics of crypto users. First, a lot of dishonesty we hear about the crypto space is associated with business (vs. investors) that offer fraudulent coins, exchanges, and custody services that try to scam away investors’ funds. The current research focuses on understanding the larger group of people who are interested in adopting and investing in crypto rather than a small number of potentially fraudulent businesses. Second, while early adopters of bitcoin used bitcoins for the exchange of illicit products, the defining characteristic of cryptocurrency that records every transaction on openly-accessible blockchain makes cryptocurrency a terrible means for dishonest activities. With this knowledge more publicly available at the current stage of adoption, we do not think dishonesty is, and will be, the crucial defining characteristic of crypto adopters as the adoption grows and the public is better informed. Instead, we focused on understanding the moral foundation of crypto advocates because moral foundations offer stronger basis of one’s attitudes and behavior than many other personality variables (Skitka et al., 2005; Skitka and Bauman, 2008).

Another interesting path for future crypto research is to investigate the different characteristics of people who adopt crypto as store of wealth (or means of invest) vs. medium of exchange. At the current stage of adoption with limited outlets to spend crypto to make a purchase, most people adopt crypto as a speculative investment. Moreover, additional research may examine the interplay between moral foundations and specific behaviors in interacting with cryptocurrency (e.g., HODLing vs. day trading)—for instance, by examining differences in trading behaviors among investors who hold more binding vs. individualizing moral foundations themselves. As crypto adoption grows across the world and an increasing number of businesses accept crypto payment, researchers will have the opportunity to further understand the effect of different motivations in crypto adoption.

As crypto technologies increasingly have a growing impact on payments, investments, and financial decision making behavior, it is increasingly important to understand and preempt the risks and vulnerabilities people may encounter within this new domain. This work seeks to provide a perspective through moral foundations and political ideology that we hope will spur further research in this effort.

## Data availability statement

The raw data supporting the conclusions of this article will be made available by the authors, without undue reservation.

## Ethics statement

The ethics protocols used for the study involving human participants were reviewed and approved by University of Utah IRB. The participants provided their informed consent to participate in this study through the online platform where the study was conducted.

## Author contributions

All authors listed have made a substantial, direct, and intellectual contribution to the work and approved it for publication.

## Conflict of interest

The authors declare that the research was conducted in the absence of any commercial or financial relationships that could be construed as a potential conflict of interest.

## Publisher’s note

All claims expressed in this article are solely those of the authors and do not necessarily represent those of their affiliated organizations, or those of the publisher, the editors and the reviewers. Any product that may be evaluated in this article, or claim that may be made by its manufacturer, is not guaranteed or endorsed by the publisher.
